# Vinylogous Mukaiyama aldol reactions with 4-oxy-2-trimethylsilyloxypyrroles: relevance to castanospermine synthesis

**DOI:** 10.1186/1860-5397-3-38

**Published:** 2007-11-03

**Authors:** Roger Hunter, Sophie C M Rees-Jones, Hong Su

**Affiliations:** 1 Department of Chemistry, University of Cape Town, Private Bag, Rondebosch, Cape Town 7701, South Africa

## Abstract

**Background:**

The diastereoselectivity of a vinylogous Mukaiyama aldol reaction of a series of *N*-substituted 4-oxy-2-trimethylsilyloxypyrroles with a tartrate-based aldehyde has been explored as a model reaction for castanospermine synthesis.

**Results:**

The study has revealed that the reaction is sensitive to the nature of the combination of *N*- and 4-oxy substituents. With a *N*-PMB or *N*-Bn and 4-methoxy combination, the reaction generates an aldol adduct with the correct absolute configurations for C-8 and C-8a of the indolizidine alkaloid castanospermine. The adduct was transformed to an indolizidine, whose ketal could not be transformed appropriately for the target alkaloid.

**Conclusion:**

The first successful diastereoselective Mukaiyama aldol strategy for the C-8 and C-8a stereogenic centres of castanospermine is presented using silyloxypyrrole chemistry. The results suggest that a full enantioselective synthesis can be realized provided that C-1 functionalisation is accomplished early in the synthesis, post-coupling.

## Background

**Scheme 1:**
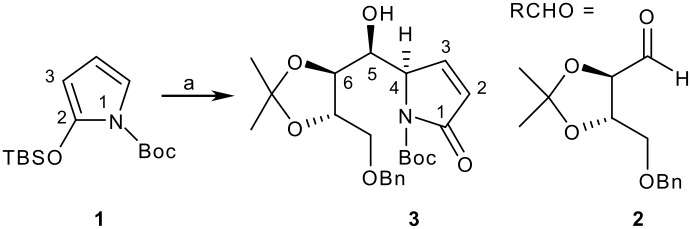
An example of a vinylogous Mukaiyama aldol in a natural product synthesis. *Reagents and conditions*: a) RCHO **2**, SnCl_4_ (1.2 equiv), ether, -85°C.

*N*-Protected silyloxypyrroles have emerged in recent years as powerful synthetic building blocks for synthesis, particularly of pyrrolizidine and indolizidine alkaloids.[1–3] Following the pioneering work of Casiraghi, *N*-(*t*-Boc)-2-(*t*-butyldimethylsilyloxy)pyrrole TBSOP **1** has established itself as the reagent of choice for promoting extended (vinylogous) Mukaiyama addition reactions to aldehydes,[4] imines [5] and conjugatively to enones [6–7] under Lewis-acid mediated dissociative reaction conditions. Many of these reactions reveal high diastereoselectivities, which has been exploited to access a range of natural products and their derivatives.[8–9] An example of Casiraghi's from the castanospermine repertoire showing the numbering system used in this article is shown in Scheme 1.[4]

While TBSOP **1** can be prepared, isolated and stored (at low temperature) for general use, its synthesis involves usage of the relatively expensive TBSOTf. From the outset of this work, we were interested in developing a cost-effective alternative using TMS rather than TBS as the silylating source and changing the *N*-Boc group to Bn or PMB. As expected, such changes precluded isolation of the silyloxypyrrole and resulted in us developing a one-pot methodology involving its *in situ* generation. We have recently demonstrated the applicability of *N*-protected-4-methoxy-2-trimethylsilyloxy pyrroles **5** to the synthesis of key intermediates for the alkaloids lepadiformine [10] and castanospermine [11] using vinylogous Mukaiyama aldol reactions. For the latter, silyloxypyrrole **5a** was shown to give adduct **6a** with the correct stereogenicities at C-4 and C-5 (C-8a and C-8 respectively in the alkaloid) for castanospermine.

**Scheme 2:**
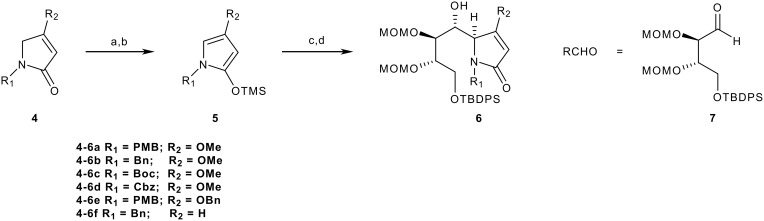
Our vinylogous Mukaiyama aldol reaction with different substituents. *Reagents and conditions*: a) *n*-BuLi, (1.5 equiv), THF, -78°C, 30 mins; b) TMSCl (3 equiv), -78°C, 30 mins; c) **7** (0.7 equiv), THF, -78°C; d) SnCl_4_ (2 equiv), -78°C to -20°C.

This paper reports on the influence of substitution changes in **5** (R_1_ and R_2_) on the outcome of extended Mukaiyama reactions with aldehyde **7**, and demonstrates how one of the adducts can be transformed into an advanced intermediate for castanospermine synthesis (Scheme 2).

## Results and Discussion

### Changing the N-protecting group

**Figure 1:**
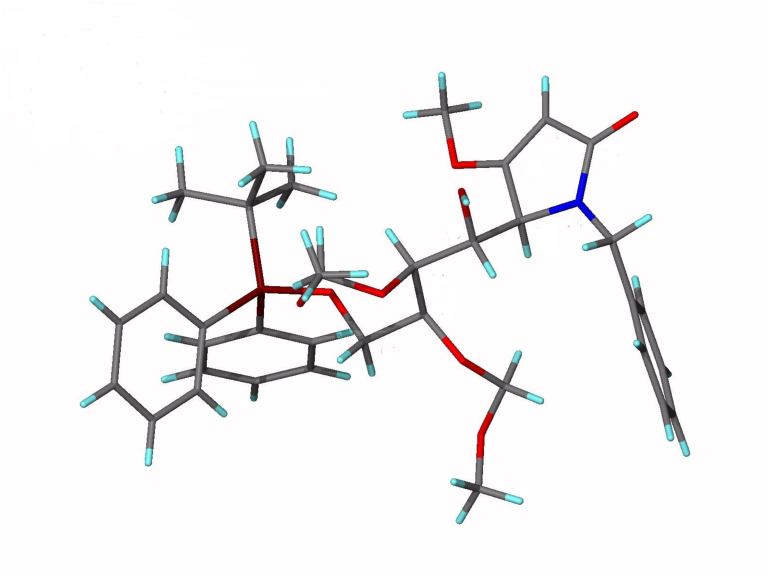
X-ray crystal structure of **6b**.

Synthesis of pyrrolinones **4a** and **4b** was straightforward involving condensing the appropriate amine (PMBNH_2_ or BnNH_2_ respectively) with ethyl *E*-4-chloro-3-methoxybut-2-enoate.[11] Reaction of **4b** with *n*-BuLi as before followed by TMSCl to generate **5b**, and reaction with aldehyde **7** (0.7 equiv) as limiting reagent using SnCl_4_ (2 equiv) as the Lewis-acid promoter with rapid stirring of the reaction gave a single crystalline diastereomer **6b** as the major product (57%) together with a mixed fraction (33%) following chromatography. The latter revealed a complex array for the H-4 and H-5 signals in its ^1^H NMR spectrum, and no conclusive stereochemical assignments could be made. By comparison and as with **6a**,[11] the key NMR signals of H-4 and H-5 in the ^1^H NMR and C-4 and C-5 in the ^13^C NMR of **6b** suggested an identical stereochemistry to that of adduct **6a**, and this was unambiguously confirmed for **6b** as the 4,5-*erythro*-5,6-*threo* adduct by a single crystal X-ray determination (Figure 1). CCDC 653714 contains the Supplementary Crystallographic Data for compound **6b**. These data can be obtained free of charge from the Cambridge Crystallographic Data Centre via http://www.ccdc.cam.ac.uk/data_request/cif. Such a result confirmed the unimportance of the *p*-methoxy group of the *N*-PMB protecting group on the diastereoselectivity of the reaction. Given that tlc indicated that reaction only begins at around -50°C, and that the reaction is quenched at -20°C, we believe the major adduct to be the kinetic product. However, a more comprehensive study is needed to support this view beyond reasonable doubt.

Attention was then turned to changing the *N*-protecting group to a carbamate in line with TBSOP **1**. The pyrroline precursor **4** (R_1_ = H, R_2_ = OMe) was readily prepared via condensation of ethyl *E*-4-chloro-3-methoxybut-2-enoate with ammonia, and then transformed to carbamates **4c** and **4d** under the standard conditions of (Boc)_2_O/DMAP and NaH/CBzCl respectively. Each was independently subjected to our *in situ* Mukaiyama sequence involving *n*-BuLi followed by TMSCl, and then addition of aldehyde **7** and SnCl_4_. However, following the normal work-up, tlc analysis in each case revealed consumption of starting pyrrolinone with formation of a multitude of products. This was attributed to the instability of the pyrrolinone carbamates **4c** and **4d** towards *n*-BuLi, and indicated the need to resort to the Casiraghi conditions of 2,6-lutidine and TBSOTf for silyloxypyrrole generation.[12] However, in view of our objective of developing a cost-effective method, this option was not pursued.

### Changing the 4-oxy substituent

In terms of synthetic design, the 4-methoxy substituent was envisaged as having implications for reactivity of the silyloxypyrrole as well as relevance to installation of the C-1 hydroxyl in a convergent castanospermine synthesis using a Mukaiyama aldol reaction as a key step. Thus it was decided to investigate the influence of changing both the O-protecting group as well as substituting the 4-oxy substituent with hydrogen. Deprotection of a methyl ether to its hydroxyl group requires aggressive conditions that can present problems in end-game aspects of total synthesis. Thus it was decided to change methyl to the more deprotection-friendly benzyl group. Synthesis of benzyl pyrrolinone **4e** was readily achieved by heating **4a** with excess benzyl alcohol at 80°C in an acid-catalysed (*p*-TsOH) exchange with *in vacuo* (water pressure) removal of methanol as it formed. Thus **4e** was isolated in 65% yield after chromatography. Subjecting **4e** to the standard silyloxypyrrole formation conditions as before to afford **5e** *in situ* followed by reaction with aldehyde **7** resulted in isolation of a major diastereoisomeric adduct in 63% yield following conventional work-up and chromatography. Although an X-ray structure determination was not carried out, the tlc and spectral characteristics provided strong evidence that adduct **6e** had the same C_4/5_ stereochemistry as **6a, b**. For example, the coupling constant between H-5/H-6 of **6e** of 6.0 Hz compared to 7.2 and 7.3 Hz for **6a** and **6b** respectively indicated the same relative 5/6 stereochemistry (*threo*)[9] as in **6a/b**. Unfortunately, the signals for H-4 and H-5 were part of a complex signal and thus ascertaining the relative stereochemistry at H-4 was more difficult. However, the [α]_D_ found in this case had a positive value, as it was for **6a** and **6b**. Casiraghi has demonstrated that the sign of the rotation is dependent on C-4 absolute configuration,[4] and it is likely that the same holds here, thus since the ^13^C values of **6e** for C-4, C-5 and C-6 were similar within 1 ppm to those for **6a** and **6b**, the absolute stereochemistries at C-4 and C-5 of **6e** were taken as likely to be the same as those for **6a** and **6b**.

**Scheme 3:**
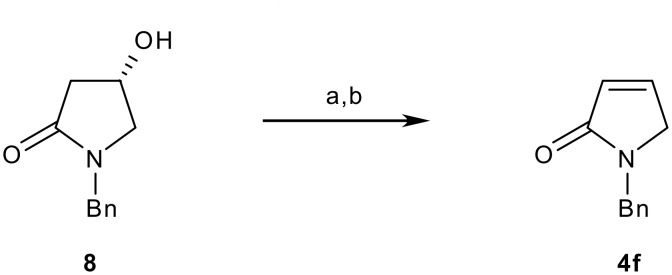
Synthesis of *N*-benzyl-3-pyrrolin-2-one **4f**. *Reagents and conditions*: a) MsCl, Et_3_N, DMAP, 90%; b) Et_3_N, THF, Δ, 70%.

Finally, the role of the C-4 methoxy group was investigated. Thus, pyrrolinone **4f** (R_2_ = H) was prepared from the known 4-hydroxy lactam **8** [13–15] via elimination of its mesylate (Scheme 3). Subsequent to this we became aware of a much shorter sequence for realising **4f** via condensation of dimethoxydihydrofuran with benzylamine [16] or *via* a ring-closing metathesis reaction.[17]

Pyrrolinone **4f** was then subjected to the standard Mukaiyama aldol sequence (Scheme 2), but tlc indicated that no reaction to form an aldol adduct had taken place. The reaction sequence was repeated and each time starting pyrrolinone was recovered. Assuming that formation of the silyloxypyrrole occurred in this sequence, which we feel to be likely in this case, this outcome came as an interesting and unexpected result, particularly since chiral *N*-alkyl silyloxypyrroles are known to undergo asymmetric vinylogous Mukaiyama aldol reactions with simple aldehydes.[3,18] It would appear that in our case in the *N*-benzyl series, a C-4 methoxy group is essential for reactivity by raising the energy of the silyloxypyrrole HOMO. However, this still leaves the question of why Casiraghi's TBSOP **1** with an *N*-carbamate protecting group is so effective compared to our case **5f** with the *N*-benzyl group in which the nitrogen lone pair is more delocalized into the pyrrole ring. Possible interference of the Lewis-acid at the nitrogen of **5f** (not likely in TBSOP) is a possible explanation, which presumably changes in the presence of the methoxy group of **5a**. The latter possibility is in line with the transition-state model recently postulated by us in which it was suggested that the methoxy group plays a coordinating role in the extended Mukaiyama aldol addition resulting in an *endo*-like transition state.[11] In the known cases [3,18] of *N*-alkyl silyloxypyrroles successfully reacting with simple aldehydes just mentioned, a more hindered α-asymmetric benzyl centre on nitrogen was used which would have presented steric hindrance at nitrogen towards the Lewis-acid.

We have also previously reported on the use of 4-methoxy-2-trimethylsilyloxypyrroles for generating C-5 quaternary centres using trimethyl orthoformate as the electrophile and BF_3_·OEt_2_ as the promoter.[10] Together with the present study, this confirms the usefulness of *N*-benzyl-4-oxy-2-trimethylsilyloxypyrroles in vinylogous Mukaiyama aldol reactions.

**Figure 2:**
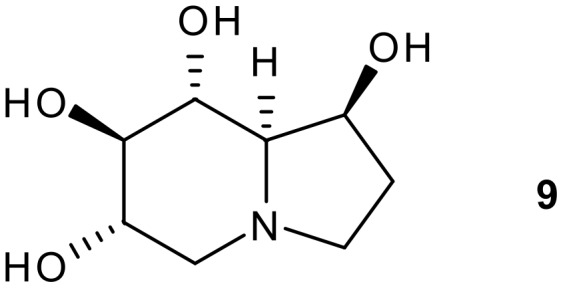
Structure of (+)-castanospermine **9**.

**Figure 3:**
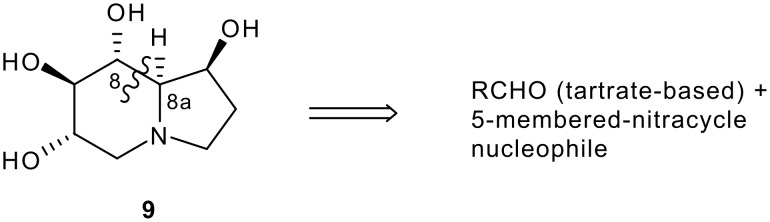
C-8/C-8a disconnection stategy for castanospermine synthesis.

As a demonstration of the usefulness of the methodology described herein, adduct **6a** was transformed into an advanced intermediate for the synthesis of (+)-castanospermine **9** (Figure 2). The latter is an indolizidine alkaloid that has received significant attention from the synthetic organic community in view of its potent biological activity as an α- and β-glycosidase inhibitor with promising anti-diabetic,[19] anti-cancer,[20] anti-viral [21] and anti-AIDS activity.[22] The early castanospermine syntheses were carbohydrate-based, and used glucose or mannose as their starting materials. However, in more recent times there has been a trend towards using other building blocks from the chiral pool to introduce some of the chiral centres.[23–24] Of these approaches, a number have utilized a convergent stategy via a C-8/C-8a disconnection (Figure 3). Thus, Gallagher,[25] Martin [26–27] and Casiraghi [4] have all attempted syntheses involving either a carbanion at C-8a or a Lewis-acid promoted aldol reaction as in Scheme 1 to form the C-8/C-8a bond. All the syntheses have suffered from incorrect diastereoselectivity in the aldol step as well as a lack of provision for C-1 hydroxyl group installation, and none of the syntheses based on this disconnection to date have successfully synthesized the target **9**. Given our success [9] in synthesizing the 4,5-*erythro*-5,6-*threo* adduct **6a** (Scheme 2) with correct absolute configurations for castanospermine (C-8/C-8a), we sought to transform **6a** into the target alkaloid, and in this paper report on the synthesis of an advanced intermediate towards this goal.

**Scheme 4:**
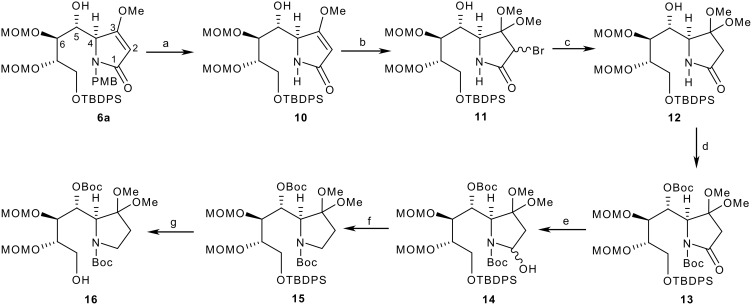
Synthesis of Adduct **16** from adduct **6a**. *Reagents and conditions*: a) CAN, aq CH_3_CN, -20°C to rt, 5 h, 82%; b) Br_2_, MeOH, -20°C, 30 mins, 91%; c) Zn, aq NH_4_Cl, THF, MeOH, RT, 30 mins, 90%; d) (Boc)_2_O (4 equiv), THF, DMAP (cat), rt, 18 h, 90%; e) DIBAL-H, THF, -78°C to -20°C, 2 h, 86%; f) Et_3_SiH, BF_3_·OEt_2_, DCM, -70°C, 1 h, 91%; g) TBAF, THF, 10°C, 5 days, 84%.

Thus, adduct **6a** underwent oxidative cleavage of the PMB protecting group under acid conditions with CAN to give lactam **10** in 82% yield with retention of the MOM groups (Scheme 4). The vinyl ether **10** was then converted to a ketal in order to preclude any epimerization occurring at C-4, and this was achieved by subjecting **10** to bromine in the presence of methanol to give bromoketal **11** in 91% yield as a mixture of diastereomers (4:1). The diastereomeric mixture of **11** then underwent zinc reduction in the presence of methanol/THF/ammonium chloride [28] to give ketal **12** in 90% yield as a single diastereomer as evidenced by ^1^H and ^13^C NMR spectra. Having developed C-1 functionality in a protected form, attention turned to lactam carbonyl group removal and cyclization. In order to avoid interference during cyclization from the C-5 hydroxyl group as well as to link up with the lactam reduction methodology, the C-5 hydroxyl group and lactam N-H were both converted to their Boc derivatives using standard conditions ((Boc)_2_O (4 equiv), THF, DMAP) to give **13** in 90% yield as a crystalline solid. *N*-Boc lactam **13** was then reduced to lactol **14** with DIBAL-H to afford a mixture of diastereomers in 86% yield, which was reduced with triethylsilane in the presence of BF_3_·OEt_2_ to give carbamate **15** in 91% yield. This was then desilylated with TBAF at 10°C for 5 days to give alcohol **16** in 84% yield. These conditions were chosen, as by-products formed at higher temperature.

**Scheme 5:**
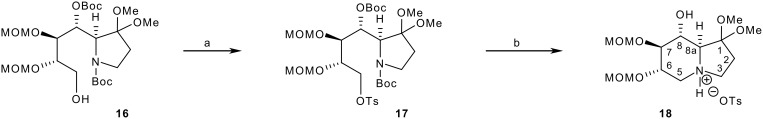
Conversion of adduct **16** to indolizidine **18**. *Reagents and conditions* a) (1.5 equiv), Et_3_N (2 equiv), DMAP (cat), DCM, 27°C, 24 h, 100%; b) i) TFA: DCM (1:4), 0°C 2 h; ii) Hünig's base (4 equiv), DCM, 0°C, 18 h, 56%.

**Figure 4:**
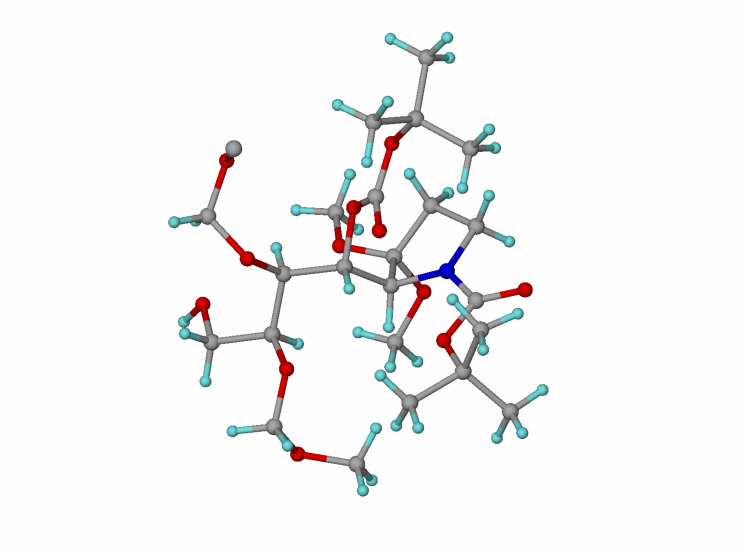
X-ray structure of **16**.

Adduct **16** was crystallized from ethyl acetate and hexane and used to obtain a single-crystal X-ray structure (Figure 4). CCDC 653715 contains the Supplementary Crystallographic Data for compound **16**. These data can be obtained free of charge from the Cambridge Crystallographic Data Centre via http://www.ccdc.cam.ac.uk/data_request/cif. The structure revealed the tartrate-derived centres C-6 and C-7 to be in their correct absolute configurations as derived from L-tartrate (both *S*-), and thus established the C-4 and C-5 absolute configurations to be *S*-, and *R*- respectively as shown in Scheme 4 and correct for castanospermine synthesis. The result also confirmed that the Mukaiyama adduct **6a** had not epimerized during this end-game sequence. Interestingly, the *N*-Boc group appears in its s-*trans* form, with the carbonyl oxygen pointing away from C-4. Compound **16** was then converted in high yield to tosylate **17** for the final cyclization, hydrolysis and reduction sequence to target (Scheme 5).

Exposure of **17** to TFA at 0°C followed by addition of Hünig's base resulted in cyclization to indolizidine **18**, which was isolated chromatographically as a tosylate salt in 56% yield. Compound **18** was identified from its NMR data. In particular, C-3(H-3), C-5(H-5) and C-8a(H-8a) resonated ≈ 10 ppm (C) and 1 ppm (H) downfield respectively compared to the signals in castanospermine as a result of deshielding by the quaternised nitrogen atom. Indolizidine **18** indicated concomitant hydrolysis of both MOM ethers to have occurred. A minor, less polar fraction was identified as a partially protected (C-6 or C-7) indolizidine (26%) that could independently be transformed into **18** by treatment with HCl. Unfortunately, attempts to hydrolyse the C-1 ketal of **18** to its carbonyl function under a variety of concentrations of acid (HCl) and at different temperatures all failed. Prolonged treatment led to decomposition. We attribute this unreactivity to destabilization of the intermediate oxocarbenium ion by the adjacent α-ammonium cation.

## Conclusion

In summary, the present work has laid the foundation for a full enantioselective synthesis of castanospermine using a C-8/C-8a disconnection strategy. Future work will focus on C-1 transformation earlier in the sequence.

## Experimental

**Figure 5:**
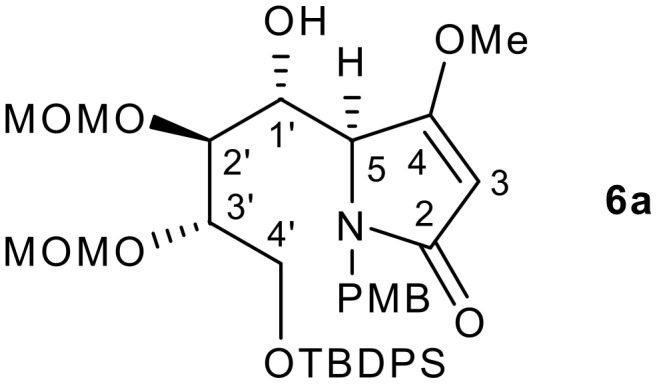
Structure of **6a** with numbering to demonstrate numbering system used in this section.

See Supporting Information File 1 for full experimental data. Figure 5 describes the numbering system used in the experimental section.

## Supporting Information

File 1 **General methods and Experimental**. Experimental details for compounds **4** to **18**. Contains Figure 5.
