# Supplementary material for: Vinylogous Mukaiyama aldol reactions with 4-oxy-2-trimethylsilyloxypyrroles: relevance to castanospermine synthesis
Source: Beilstein J Org Chem. 2007 Nov 3;3:38. doi: 10.1186/1860-5397-3-38 (PMC2151077; doi:10.1186/1860-5397-3-38)
Supplement: File 1 — General methods and Experimental. Experimental details for compounds 4 to 18. Contains Figure 5. [file Beilstein_J_Org_Chem-03-38-s001.doc]

**General methods**

Melting points were obtained using a Reichert-Jung Thermovar hot-stage microscope and are uncorrected. Elemental analyses were performed using a Fisons EA 1108 CHNS elemental analyzer. Infra-Red (IR) absorptions were measured on a Perkin Elmer Spectrum One FT-IR Spectrometer. Nuclear Magnetic Resonance spectra were recorded on a Varian Mercury 300 MHz (75.5 MHz for ^13^C) or Varian Unity 400 (100.6 MHz for ^13^C) instrument and were carried out in chloroform-d unless otherwise stated. Chemical shifts (δ) were recorded relative to residual chloroform (δ 7.26 in ^1^H NMR) and (δ 77.00 in ^13^C NMR). All chemical shifts are reported in ppm and resonances are assigned according to IUPAC numbering, viz H-1 = H on C-1. Optical rotations were obtained using a Perkin Elmer 343 polarimeter at 20°C. The concentration *c* refers to g/100ml. High resolution mass spectra were recorded on a Waters API Q-TOF Ultima machine at the Mass Spectrometry Service, School of Chemistry University of Stellenbosch.

Single crystal X-ray intensity data were collected on a Nonius Kappa-CCD diffractometer using graphite monochromated MoKα radiation. The temperature was controlled by an Oxford Cryostream cooling system (Oxford Cryostat). The strategy for the data collections was evaluated using the Bruker Nonius "Collect” program. Data were scaled and reduced using DENZO-SMN software (Otwinowski & Minor, 1997).

Both structures were solved by direct methods and refined employing the full-matrix least-squares method with the program SHELXL-97 refining on F^2^. Packing diagrams were produced using the program PovRay and graphic interface X-seed. All non-H atoms were refined anisotropically. ALL hydrogen atoms except the hydroxyl hydrogens were placed geometrically and were refined using a riding model, with C–H = 0.95 – 1.00 Å and U_iso_ = 1.2 -1.5 x U_eq_(C). The hydroxyl hydrogens were located in the difference electron-density maps and refined with simple bond-length constraints. Both structures were refined successfully with R = 0.0553 for **6b** and R = 0.0472 for **21**.

**Experimental**

4-*O*-*tert*-Butyldiphenylsilyl-2,3-*O*-di-methoxymethyl-L-threitol

To a solution of 2,3-Di-*O*-methoxymethyl-L-threitol [29] (6.0 g, 28.5 mmol) at 0°C in dry tetrahydrofuran (300 ml), was added *n*-butyllithium (1.6M in hexane, 17.9 ml, 28.5 mmol, 1 eq) drop-wise via a syringe. The reaction mixture was stirred at 0°C for 40 minutes. *tert-*Butyldiphenylsilyl chloride (7.5 ml, 28.5 mmol, 1 eq) was then added drop-wise and the reaction mixture was stirred for a further hour at 0°C followed by 18h at room temperature. The reaction mixture was quenched with aqueous sodium hydrogen carbonate (200 ml) and was concentrated under reduced pressure. The solution was extracted with dichloromethane (3 x 200 ml) and with ethyl acetate (2 x 200 ml). The combined organic layers were dried over magnesium sulfate and concentrated to yield a residue (16.5 g), which was chromatographed on silica-gel (140 g) with ethyl acetate / petroleum ether mixtures (1 : 9 to 3 : 2), as eluent to yield the titled silyl ether (12.0 g, 26.9 mmol, 94%).

[α]_D_ +1.5 (*c* 1.5, CH_2_Cl_2_); ([M+H]^+^ Found 448.2300, C_24_H_36_O_6_Si requires 448.2281.); υ_max_ /cm^-1^ 3466 (O-H), 3071, 3054 (C-H arom), 2985, 2954, 2933, 2892, 2859, 2826, (C-H aliph),1149, 1112 (C-O ether); δ_H_ (CDCl_3_, 400 MHz) 1.06 (9H, s, *t*-Bu), 3.10 (1H, t, *J* 6.8 Hz, OH), 3.34 (3H, s, OCH_3_), 3.37 (3H, s, OCH_3_), 3.70-3.90 (6H, m, H-1,4 and H-2,3), 4.61 (1H, d, *J*_AB_ 6.8 Hz, OCH_2_O), 4.64 (1H, d, *J*_AB_ 7.0 Hz, OCH_2_O), 4.68 (1H, d, *J*_AB_ 7.0 Hz, OCH_2_O), 4.68 (1H, d, *J*_AB_ 6.8 Hz, OCH_2_O), 7.40 (6H, m, Ar), 7.66 (4H, m, Ar); δ_C_ (CDCl_3_, 100.6 MHz) 19.1 (Si-C), 26.8 ((CH_3_)_3_), 55.8 (OCH_3_), 55.9 (OCH_3_), 62.7 (C-1, C-4), 63.0 (C-1, C-4), 78.1 (C-2, C-3), 80.5 (C-2, C-3), 97.2 (OCH_2_O), 97.7 (OCH_2_O), 127.7, 127.7, 129.8, 129.8, 133.1, 133.2, 135.5, 135.5 (Ar);

4-*O*-*tert*-Butyldiphenylsilyl-2,3-*O*-di-methoxymethyl-L-threose **7**

To a solution of dimethyl sulfoxide (1.6 ml, 22 mmol, 3 eq) in dry dichloromethane (30 ml) at -78°C, was added oxalyl chloride (1.3 ml, 14.8 mmol, 2 eq) drop-wise via a syringe. The reaction mixture was stirred for 10 minutes at -78°C. 4-*O*-*tert*-Butyldiphenylsilyl-2,3-*O*-di-methoxymethyl-L-threitol (3.3 g, 7.4 mmol) in dry dichloromethane (10 ml) was then added to the mixture drop-wise, followed by triethylamine (5.2 ml, 37 mmol, 5 eq). After a further 20 minutes. The reaction mixture was allowed to warm to 0°C, upon which the reaction was quenched with sodium carbonate (100 ml) and extracted with ethyl acetate (3 x 200 ml). The combined organic layer was dried over magnesium sulfate and concentrated to yield a residue (4 g), which was chromatographed on silica-gel (80 g) with ethyl acetate / petroleum ether mixtures (1 : 9 to 1 : 1) as eluent to yield aldehyde **7** (3.0 g, 6.7 mmol, 90%).

[α]_D_ +9.5 (*c* 0.75, CH_2_Cl_2_); ([M+H]^+^ Found 447.2224, C_24_H_35_O_6_Si requires 447.2203.); υ_max_ /cm^-1^ 3070, 3054 (C-H arom), 2986, 2957, 2933, 2894, 2859, 2827 (C-H aliph), 2742 (C-H aldehyde), 1733 (C=O aldehyde), 1152, 1112 (C-O ether); δ_H_ (CDCl_3_, 300 MHz) 1.05 (9H, s, *t*-Bu), 3.23 (3H, s, OCH_3_), 3.40 (3H, s, OCH_3_), 3.81 (2H, d, *J* 6.5 Hz, H-4), 4.07 (1H, td, *J* 6.5, 3.2 Hz, H-3), 4.27 (1H, dd, *J* 3.2, 0.8 Hz, H-2), 4.52 (1H, d, *J* 6.8 Hz, OCH_2_O), 4.58 (1H, d, *J* 6.8 Hz, OCH_2_O), 4.73 (1H, d, *J* 6.9 Hz, OCH_2_O), 4.78 (1H, d, *J* 6.9 Hz, OCH_2_O) 7.30-7.50 (6H, m, Ar), 7.60-7.70 (4H, m, Ar), 9.80 (1H, d, *J* 0.8 Hz, H-1); δ_C_ (CDCl_3_, 100.6 MHz) 19.1 (Si-C), 26.8 ((CH_3_)_3_), 55.8 (OCH_3_), 56.2 (OCH_3_), 61.8 (C-4), 77.4 (C-3), 81.4 (C-2), 96.7 (OCH_2_O), 97.5 (OCH_2_O) 127.8, 127.8, 129.9, 129.9, 132.9, 133.0, 135.6, 135.6 (Ar), 202.1 (C-1).

4-Methoxy-1-(4-methoxybenzyl)-3-pyrrolin-2-one **4a**

To a solution of *para*-methoxybenzylamine (4.8 ml, 36 mmol, 1.2 eq) in dry acetonitrile (70 ml) at 0°C, was added Hünig’s base (7.7 ml, 46 mmol, 1.5 eq) drop-wise via a syringe followed by methyl 4-chloro-3-methoxy-(*E*)-2-butenoate (4.1 ml, 30 mmol). The reaction mixture was heated with stirring at 60°C for 18 hrs. The reaction was quenched with saturated sodium hydrogen carbonate (150 ml) and extracted with ethyl acetate (3 x 200 ml). The combined organic layer was dried over magnesium sulfate and concentrated to yield a residue (6 g), which was chromatographed on silica-gel (120 g) with ethyl acetate / petroleum ether mixtures (3 : 7 to 7 : 3) as eluent to yield **4a** (4.7 g, 20 mmol, 67%).

M.p. 73-74°C (from ethyl acetate-hexane); (Found: C, 66.82; H, 6.42; N, 5.65%. C_13_H_15_NO_3_ requires C, 66.94; H, 6.48; N, 6.00%); ([M+H]^+^ Found 234.1128, C_13_H_16_NO_3_ requires 234.1130.); υ_max_ /cm^-1^ 2929, 2838 (C-H arom), 1671 (C=O), 1624 (C=C), 1115 (C-O); δ_H_ (CDCl_3_, 300 MHz) 3.68 (2H, s, H-5), 3.74 (3H, s, OCH_3_), 3.77 (3H, s, OCH_3_), 4.48 (2H, s, Bn), 5.06 (1H, s, H-3), 6.83 (2H, d, *J*_AB_ 8.7 Hz, Ar), 7.14 (2H, d, *J*_AB_ 8.7 Hz, Ar). δ_C_ (CDCl_3_, 75.5 MHz) 44.7 (Bn), 49.7 (C-5), 55.2 (OCH_3_), 58.0 (OCH_3_), 94.2 (C-3), 114.1, 129.2, 129.5, 159.0 (Ar), 172.0 (C-4), 173.3 (C-2).

1’*R*, 2’*S*, 3’*S*, 5*S*, 5-[4’-*tert*-butyldiphenylsilyloxy-1-hydroxy-2’,3’-bis-methoxymethoxy]-butyl-4-methoxy-1-(4-methoxybenzyl)-3-pyrrolin-2-one **6a**, (Figure 5).

To a solution of pyrrolinone **4a** (0.39 g, 1.7 mmol) in dry tetrahydrofuran (5.0 ml) at -78°C, was added *n*-butyllithium (1.6 M in hexane, 1.6 ml, 2.5 mmol, 1.5 eq) drop-wise via a syringe. The reaction mixture was stirred for 30 minutes. Trimethylsilyl chloride (0.64 ml, 5.0 mmol, 3 eq) was added drop-wise and the reaction mixture was stirred for a further 30 minutes. Aldehyde **7** (0.55 g, 1.2 mmol) in dry tetrahydrofuran (4.0 ml) was added drop-wise followed by tin (IV) chloride (1M in dichloromethane, 2 ml, 2.0 mmol) drop-wise. The reaction mixture was stirred vigorously and allowed to warm up to -20°C over three hours, after which it was stirred at -20°C for 18 hours. The reaction mixture was quenched with cold saturated sodium hydrogen carbonate (30 ml) and extracted with ethyl acetate (3 x 50 ml). The combined organic layer was washed with brine (50 ml) and dried over magnesium sulfate. The organic layer was concentrated under reduced pressure to yield a residue (1.2 g), which was chromatographed on silica-gel (65 g) with ethyl acetate / petroleum ether mixtures (3 : 7 to 7 : 3) as eluent to give diastereomer **6a** (0.52 g, 0.76 mmol, 63%), mixed diastereomers (0.20 g, 0.29 mmol, 24%), and unreacted aldehyde **7** (0.06 g, 0.13 mmol, 11%).

Major- **6a** M.p. 107-108°C (from ethyl acetate-hexane); [α]_D_ = +6.67 (c 3.0, CH_2_Cl_2_); (Found: C, 65.52; H, 7.36; N, 2.10%. C_37_H_49_NO_9_Si requires C, 65.37; H, 7.26; N, 2.06%.); ([M+H]^+^ Found 680.3280, C_37_H_50_NO_9_Si requires 680.3255.); υ_max_/cm^-1^ 3053 (OH), 2933 (CH), 1678 (C=O), 1628 (C=C); δ_H_ (CDCl_3_, 300 MHz) 1.06 (9H, s, *t*-Bu), 3.08 (3H, s, OCH_3_), 3.34 (3H, s, OCH_3_), 3.68 (3H, s, OCH_3_), 3.74 (3H, s, OCH_3_), 3.75-3.90 (3H, m, H-3’, H-4’), 3.99 (1H, dd, *J* 7.1, 1.8 Hz, H-2’), 4.08 (1H, d, *J* 2.0 Hz, H-5), 4.08 (1H, d, *J* 15.3 Hz, Bn), 4.19 (1H, dd, *J* 7.1, 2.0 Hz H-1’), 4.22 (1H, d, *J* 6.5 Hz, OCH_2_O), 4.34 (1H, d, *J* 6.5 Hz OCH_2_O), 4.68 (2H, s, OCH_2_O) 5.10 (1H, d, *J* 15.3 Hz, Bn), 5.20 (1H, s, H-3), 6.79 (2H, d, *J* 8.7 Hz, PMB), 7.17 (2H, d, *J* 8.7 Hz, PMB), 7.30-7.50 (6H, m, Ar), 7.60-7.70 (4H, m, Ar); δ_C_ (CDCl_3_, 75.5 MHz) 19.2 (Si-C), 26.8 ((CH_3_)_3_), 42.8 (C-Bn), 55.2 (OCH_3_), 55.5 (OCH_3_), 56.3 (OCH_3_), 58.1 (OCH_3_), 60.9 (C-5), 62.4 (C-4’), 68.3 (C-1’), 77.9 (C-3’), 80.9 (C-2’), 95.7 (C-3), 96.8 (OCH_2_O), 99.2 (OCH_2_O), 114.0 (PMB), 127.7, 127.8, 129.2, 129.8, 129.9, 130.0, 133.0, 133.1, 135.4, 135.5 (Ar), 158.9 (PMB), 171.9 (C-4), 173.3 (C-2)

1-Benzyl-4-methoxy-3-pyrrolin-2-one **4b** [30,31]

To a solution of benzylamine (8.0 ml, 72.0 mmol, 1.2 eq) in dry acetonitrile (100 ml) at 0°C, was added Hünig’s base (9.6 ml, 90.0 mmol, 1.5 eq) drop-wise via a syringe followed by methyl 4-chloro-3-methoxy-(*E*)-2-butenoate (8.2 ml, 60.0 mmol). The reaction mixture was heated with stirring at 60°C for 18 hrs. The reaction mixture was quenched with saturated aqueous sodium hydrogen carbonate (150 ml) and extracted with ethyl acetate (3 x 250 ml). The combined organic extracts were dried over magnesium sulfate and concentrated to yield a residue (16 g), which was chromatographed on silica-gel (200 g) with ethyl acetate / petroleum ether mixtures (3 : 7 to 7 : 3) as eluent to yield the pyrrolinone **4b** (10.0 g, 49 mmol, 82%).

M.p. 43-45°C (From ethyl acetate-hexane); δ_H_ (CDCl_3_, 300 MHz) 3.68 (2H, s, H-5), 3.74 (3H, s, OCH_3_), 4.55 (2H, s, Bn), 5.05 (1H, s, H-3), 7.10-7.40 (5H, m, Ar); δ_C_ (CDCl_3_, 75.5 MHz) 45.3 (CH_2_Ph), 49.9 (C-5), 58.0 (OCH_3_), 94.1 (C-3), 127.4, 127.9, 128.7, 137.3 (Ar), 172.1 (C-4), 173.4 (C-2).

1’*R*, 2’*S*, 3’*S*, 5*S*, 1-Benzyl-5-[4’-*tert*-butyldiphenylsilyloxy-1’-hydroxy-2’,3’-bis-methoxymethoxy]-butyl-4-methoxy-3-pyrrolin-2-one **6b**

To a solution of pyrrolinone **4b** (0.32 g, 1.55 mmol) in dry tetrahydrofuran (5 ml) at -78°C, was added *n*-butyllithium (1.6 M in hexane, 1.45 ml, 2.3 mmol) drop-wise via a syringe. The reaction mixture was stirred for 30 minutes at -78°C. Trimethylsilyl chloride (0.59 ml, 4.7 mmol) was then added drop-wise and the reaction mixture was stirred for a further 30 minutes at -78°C. Aldehyde **7** (0.50 g, 1.1 mmol) in dry tetrahydrofuran (4 ml) was added, followed by tin (IV) chloride (1M in dichloromethane, 2 ml, 2.0 mmol) drop-wise. The reaction mixture was stirred vigorously, warmed up to -20°C over 3 hours, and stirred for 18 hrs at -20°C. The reaction mixture was quenched with cold saturated sodium bicarbonate (30 ml) and extracted with ethyl acetate (3 x 50 ml). The combined organic layer was washed with brine (50 ml) and dried over magnesium sulfate, before being concentrated under reduced pressure to yield a residue (0.90 g), which was chromatographed on silica-gel (65 g) with ethyl acetate / petroleum ether mixtures (3 : 7 to 6 : 4) as eluent to give diastereomer **6b** (0.41 g, 0.63 mmol, 57%), a mixture of diastereomers (0.23 g, 0.36 mmol, 33%), and unreacted aldehyde **7** (0.025 g, 0.06 mmol, 5%).

M.p. 114-115°C (from ethyl acetate-hexane); [α]_D_ +6.35 (*c* 2.0, CH_2_Cl_2_); (Found: C, 66.65; H, 7.29; N, 2.15%. C_36_H_47_NO_8_Si requires C, 66.54; H, 7.29; N, 2.16%); ([M+H]^+^ Found 650.3120, C_36_H_48_NO_8_Si requires 650.3149.); υ_max_(CH_2_Cl_2_) / cm^-1^ 3400 (OH), 1683 (C=O), 1635 (C=C); δ_H_ (CDCl_3_, 400 MHz) 1.06 (9H, s, *t*-Bu), 3.04 (3H, s, OCH_3_), 3.35 (3H, s, OCH_3_), 3.61 (1H, s, OH), 3.69 (3H, s, OCH_3_), 3.74-3.86 (3H, m, H-3’, H-4’), 4.01 (1H, dd, *J* 7.3, 1.5 Hz, H-2’), 4.09 (1H, d, *J* 1.5 Hz, H-5), 4.13 (1H, d, *J* 17.2 Hz, Bn), 4.14 (1H, d, *J*_AB_ 6.4 Hz, OCH_2_O), 4.19 (1H, dd, *J* 7.3, 1.5 Hz, H-1’), 4.25 (1H, d, *J*_AB_ 6.4 Hz, OCH_2_O), 4.68 (2H, s, OCH_2_O), 5.17 (1H, d, *J* 17.2 Hz, Bn), 5.20 (1H, s, H-3), 7.20-7.70 (15H, m, Ar); δ_C_ (CDCl_3_, 100.6 MHz) 19.2 (Si-C), 26.8 ((CH_3_)_3_), 43.3 (C-Bn), 55.5 (OCH_3_), 56.3 (OCH_3_), 58.1 (OCH_3_), 60.8 (C-5), 62.3 (C-4’), 68.2 (C-1’), 77.9 (C-3’), 80.5 (C-2’), 95.7 (C-3), 96.8 (OCH_2_O), 99.2 (OCH_2_O), 127.2, 127.7, 127.8, 127.9, 128.6, 129.8, 129.9, 133.1, 133.1, 135.4, 135.5, 137.9 (Ar), 171.9 (C-4), 173.4 (C-2);

4-Methoxy-3-pyrrolin-2-one-1-carboxylic acid *tert*-butyl ester **4c**

To a solution of pyrrolinone **4** [32] (1.00 g, 8.8 mmol) in dry acetonitrile (50 ml) at room temperature, was added di-*tert*-butyl dicarbonate (2.03 ml, 8.8 mmol, 1 eq) followed by 4-dimethylaminopyridine (0.10 g, 0.88 mmol, 0.1 eq). The reaction mixture was stirred at room temperature for 2h. The reaction mixture was concentrated under reduced pressure to give a residue (1.89 g), which was chromatographed on silica-gel (65 g) using ethyl acetate / petroleum ether (7 : 3) as eluent to give **4c** (1.58 g, 7.4 mmol, 84%).

M.p. 94-95°C (from ethyl acetate); (Found: C, 56.54; H, 7.36; N; 6.52%. C_10_H_15_NO_4_ requires C, 56.33; H, 7.09; N, 6.57%); (Found [M+H]^+^ 214.1079, C_10_H_16_NO_4_ requires 214.1079.); ν_max_ (CH_2_Cl_2_) / cm^-1^ 3041, 3005, 2985, 2940, 2911, 2877, 2826 (C-H aliph), 1779 (O-C=O), 1682 (C=O lactam), 1626 (C=C); δ_H_ (CDCl_3_, 400 MHz) 1.50 (9H, s, *t*-Bu), 3.80 (3H, s, OCH_3_), 4.14 (2H, s, H-5), 5.06 (1H, s, H-3), δ_C_ (CDCl_3_, 100.6 MHz) 28.0 (OCO_2_C(*C*H_3_)_3_), 49.1 (C-5), 58.4 (OCH_3_), 82.5 (O*C*(CH_3_)_3_), 94.9 (C-3),149.3 (CO_carb_), 168.9 (C-4), 174.5 (CO_lact_).

4-Methoxy-3-pyrrolin-2-one-1-carboxylic acid benzyl ester **4d**

To a solution of pyrrolinone **4** (0.80 g, 7.08 mmol) in dry tetrahydrofuran (50 ml) at 0°C, was added sodium hydride (60% in oil, 0.38 g, 21.2 mmol, 3 eq) and the reaction mixture stirred at 0°C for 10 minutes. Benzyl chloroformate (1.1 ml, 7.8 mmol, 1.1 eq) was added at 0°C and the reaction mixture stirred at room temperature for 3h. The reaction mixture was quenched with aqueous ammonium chloride (100 ml) and extracted with ethyl acetate (3 x 150 ml). The combined organic layer was dried over magnesium sulfate and concentrated under reduced pressure to give a residue (2.92 g), which was chromatographed on silica-gel (70 g) using ethyl acetate / petroleum ether (7 : 3) as eluant to give **4d** (1.1 g, 3.54 mmol, 50%).

M.p. 128-129°C (from ethyl acetate); (Found: C, 63.15; H, 5.00; N; 5.61%. C_13_H_13_NO_4_ requires C, 63.15; H, 5.30; N, 5.66%); (Found [M+H]^+^ 248.0922, C_13_H_13_NO_4_ requires 248.0922.); ν_max_/cm^-1^ 3056 (C-H arom), 2986, 2943, 2898, 2870, 2856 (C-H aliph), 1781 (O-C=O), 1735 (N-C=O), 1633 (C=C), 1331; δ_H_ (CDCl_3_, 300 MHz) 3.83 (3H, s, OCH_3_), 4.23 (2H, s, H-5), 5.11 (1H, s, H-4), 5.29 (2H, s, Bn), 7.30-7.50 (5H, m, Ar); δ_C_ (CDCl_3_, 100.6 MHz) 49.1 (C-5), 58.6 (OCH_3_), 67.7 (Bn), 94.7 (C-3), 128.1, 128.3, 128.5, 135.5 (Ar), 150.6 (CO_Carb_), 168.5 (C-4), 174.9 (CO_lact_).

4-Benzyloxy-1-(4-methoxybenzyl)-3-pyrrolin-2-one **4e**

To a solution of pyrrolinone **4a** (4.00 g, 17.0 mmol) in dry benzyl alcohol (32 ml), was added a catalytic amount of *p-*toluenesulfonic acid (10 mg). The reaction mixture was heated at 80°C under reduced pressure (20 mm Hg) for five hours. The reaction mixture was quenched with aqueous sodium bicarbonate (100 ml) and extracted with ethyl acetate (3 x 200 ml). The combined organic layer was dried over magnesium sulfate and concentrated under reduced pressure. The excess benzyl alcohol was removed via vacuum distillation to yield a residue. The residue was chromatographed on silica-gel (100 g) with ethyl acetate / petroleum ether mixtures (6 : 4 to 8 : 2) as eluents to yield pyrrolin-2-one **4e** (3.4 g, 11 mmol, 65%).

M.p. 108-109°C (from ethyl acetate); (Found: C, 74.10; H, 6.20; N, 4.53%. C_19_H_19_NO_3_ requires C, 73.77, H, 6.19; N, 4.53%); Found [M+H]^+^ 310.1443 C_19_H_20_N0_3_ requires 310.1443; υ_max_(CH_2_Cl_2_)cm^-1^ 1678 (C=O), 1626 (C=C); δ_H_ (CDCl_3_, 300 MHz) 3.75 (2H, s, H-5), 3.79 (3H, s, OCH_3_), 4.50 (2H, s, NBn), 4.94 (2H, s, OBn), 5.16 (1H, s, H-3), 6.85 (2H, d, *J* 8.7 Hz, PMB), 7.17 (2H, d, *J* 8.7 Hz, PMB), 7.30-7.40 (5H, m, Ar); δ_C_ (CDCl_3_, 75.5 MHz) 44.8 (NBn), 50.0 (C-5), 55.3 (OCH_3_), 73.0 (OBn), 95.4 (C-3), 114.1 (PMB), 127.9, 128.7, 128.7, 129.3, 129.5, 134.7, 159.06 (PMB), 172.0 (C-4), 172.1 (C-2)

1’*R*, 2’*S*, 3’*S*, 5*S*, 5-[4’-(*tert*-Butyldiphenylsilyloxy)-1’-hydroxy-2’,3’-bis-methoxymethoxy]-butyl-4-benzyloxy-1-(4’’-methoxybenzyl)-3-pyrrolin-2-one **6e**

To a solution of pyrrolinone **4e** (1.64 g, 5.3 mmol, 1.5 eq) in dry tetrahydrofuran (17.5 ml) at -78°C, was added *n*-butyllithium (1.6 M in hexane, 4.96 ml, 7.9 mmol, 2.25 eq) drop-wise via a syringe. The reaction mixture was stirred for 30 minutes at -78°C. Trimethylsilyl chloride (2.02 ml, 15.9 mmol, 4.5 eq) was added drop-wise and the reaction mixture was stirred for a further 30 minutes. Aldehyde **7** (1.57 g, 3.5 mmol) in dry tetrahydrofuran (14 ml) was added drop-wise, followed by tin (IV) choride (1M in dichloromethane, 7 ml, 7.0 mmol, 2 eq) which was also added drop-wise. The reaction mixture was stirred vigorously and allowed to warm to -20°C over three hours. The reaction mixture was stirred at -20°C for 18 hours. The reaction mixture was quenched with cold aqueous saturated sodium hydrogen carbonate (150 ml) and extracted with ethyl acetate (3 x 200 ml). The combined organic layer was washed with brine (200 ml) and dried over magnesium sulfate. The organic layer was concentrated under reduced pressure to yield a residue (3.50 g), which was chromatographed on silica-gel (65 g) with ethyl acetate / petroleum ether mixtures (3 : 7 to 6 : 4) as eluent to give pure diastereomer **6e** (1.66 g, 2.2 mmol, 63%), mixed diastereomers (0.66 g, 0.87 mmol, 25 %) and unreacted aldehyde **7** (0.19 g, 0.42 mmol, 12%).

**6e** M.p. 109-110°C (from ethyl acetate-hexane); [α]_D_  +17.83 (*c* 2.3, CH_2_Cl_2_); (Found: C, 68.32; H, 6.83; N, 1.85%. C_43_H_53_NO_9_Si requires C, 68.51; H, 7.07; N, 1.85%.); Found [M+H]^+^ 756.3576, C_43_H_54_NO_9_Si requires 756.3568; υ_max_/cm^-1^ 3053 (OH), 2933 (CH), 1678 (C=O), 1628 (C=C); δ_H_ (CDCl_3_, 300 MHz) 1.03 (9H, s, *t*-Bu), 3.12 (3H, s, OCH_3_), 3.29 (3H, s, OCH_3_), 3.74 (3H, s, OCH_3_ (PMB)), 3.76-3.89 (3H, m, H-4’, H-3’), 3.92 (1H, dd, *J* 6.0 and 2.1 Hz, H-2’), 4.18 (1H, d, *J* 16.2 Hz, NBn), 4.20-4.25 (2H, m, H-5, H-1’), 4.35 (1H, d, *J_AB_* 6.5 Hz, OCH_2_O), 4.47 (1H, d, *J*_AB_ 6.5 Hz, OCH_2_O), 4.60 (1H, d, *J*_AB_ 6.9 Hz, OCH_2_O), 4.63 (1H, d, *J*_AB_ 6.9 Hz, OCH_2_O), 4.92 (2H, s, OBn), 4.96 (1H, d, *J* 16.2 Hz, NBn), 5.20 (1H, s, H-3), 6.78 (2H, d, *J*_AB_ 8.7 Hz, PMB), 7.17 (2H, d, *J*_AB_ 8.7 Hz, PMB) 7.20-7.80 (15H, m, Ar); δ_c_ (CDCl_3_, 75.5 MHz) 19.1 (Si-C), 26.8 ((CH_3_)_3_), 43.4 (Bn), 55.2 (OCH_3_), 55.5 (OCH_3_), 56.2 (OCH_3_), 61.9 (C-5), 63.2 (C-4’), 68.7 (C-1’), 73.1 (OBn), 77.9 (C-3’), 78.4 (C-2’), 96.7 (C-3), 96.8 (OCH_2_O), 98.9 (OCH_2_O), 113.9 (PMB), 127.5, 127.7, 127.7, 128.6, 128.7, 129.3, 129.3, 129.8, 130.0, 133.0, 133.1, 134.6, 135.4, 135.5 (Ar), 158.8 (PMB), 172.1 (C-4), 172.1 (CO).

4*R*, *N*-Benzyl-4-methanesulfonyloxy-pyrrolidin-2-one

To a solution of hydroxylactam **8** [13,14,15] (0.55 g, 2.9 mmol) in dry dichloromethane (20 ml) at 0°C, was added triethylamine (0.8 ml, 5.8 mmol, 2 eq), followed by mesyl chloride (0.45 ml, 5.8 mmol, 2 eq) and a catalytic amount of 4-dimethylaminopyridine (10 mg). The reaction mixture was stirred for an hour, upon which it was added to iced-water. The product was extracted with dichloromethane (3 x 100 ml) and the combined organic layers dried over magnesium sulfate. Concentration gave an oil, which was purified on silica-gel (20 g) using ethyl acetate / petroleum ether (1 : 1) as eluent to give mesylate (0.71 g, 2.6 mmol, 90%).

δ_H_ (CDCl_3_, 400 MHz) 2.71 (1H, dd, *J* 17.7, 2.7 Hz, H-5), 2.87 (1H, dd, *J* 17.7, 6.6 Hz, H-5), 2.99 (3H, s, SCH_3_) 3.50 (1H, dd, *J* 12.0, 1.5 Hz, H-3), 3.64 (1H, dd, *J* 12.0, 5.4, H-3), 4.87 (2H, s, Bn), 5.27 (1H, m, H-4), 7.20-7.40 (5H, m, Ar);

*N*-Benzyl-3-pyrrolin-2-one **4f** [16,17]

To a solution of mesylate (0.71 g, 2.6 mmol) in dry tetrahydrofuran (20 ml), was added triethylamine (0.72 ml, 5.2 mmol, 2 eq) and the reaction mixture refluxed for 24 h. The reaction mixture was quenched with saturated sodium hydrogen carbonate (20 ml). The product extracted with ethyl acetate (3 x 100 ml) and the extract dried over magnesium sulfate. Concentration gave a residue, which was purified on silica-gel (30 g) to give **4f** (0.31 g, 1.8 mmol, 70 %).

δ_H_ (CDCl_3_, 400 MHz) 3.87 (2H, t, *J* 2.0 Hz, H-5), 4.63 (2H, s, Bn), 6.22 (1H, dt, *J* 6.0, 2.0 Hz, H-3), 7.04 (1H, dt, *J* 6.0, 2.0 Hz, H-4), 7.20-7.40 (5H, m, Ar); δ_C_ (CDCl_3_, 100.6 MHz) 46.0 (Bn), 52.3 (C-5), 110.8 (C-3), 127.6, 127.9, 128.0, 128.8 (Ar), 142.8 (C-4), 170 (C-2).

1’*R*, 2’*S*, 3’*S*, 5*S*, 5-[4’-*tert*-Butyldiphenylsilyloxy-1’-hydroxy-2’,3’-bis-methoxymethoxy]-butyl-4-methoxy-3-pyrrolin-2-one **10**

To a solution of **6a** (1.00 g, 1.5 mmol) in acetonitrile / water (9 : 1), (70 ml) at -20°C, was added ceric ammonium nitrate (CAN) (3.4 g, 6.0 mmol, 4 eq). The reaction mixture was allowed to warm up to room temperature and stirred for a further 3 hours. The reaction mixture was quenched with cold saturated sodium bicarbonate (100 ml) and extracted with ethyl acetate (3 x 150 ml). The combined organic layer was dried over magnesium sulfate and concentrated to yield a residue (1.3 g). The residue was chromatographed on silica-gel (60 g) with ethyl acetate (500 ml) followed by methanol / ethyl acetate (1 : 9) as eluent to yield **10** (0.69 g, 1.2 mmol, 82%).

[α]_D_ –2.75 (c 2.0, CH_2_Cl_2_*)*; ([M+H]^+^ Found 560.2678, C_29_H_42_NO_8_Si requires 560.2680.); υ_max_ /cm^-1^ 3330 (O-H, N-H), 3071, 3051, 3014 (C-H arom), 2934, 2892, 2824 (C-H aliph), 1671 (C=O), 1622 (C=C), 1153,1110 (C-O); δ_H_  (CDCl_3_, 400 MHz) 1.05 (9H, s, *t*-Bu), 3.10 (1H, d, *J* 2.8 Hz, OH), 3.29 (3H, s, OCH_3_), 3.39 (3H, s, OCH_3_), 3.81 (3H, s, OCH_3_), 3.81 (2H, m, H-4’), 4.05 (3H, m, H-2’, H-3’ and H-5), 4.27 (1H, dd, *J* 6.8, 1.6 Hz, H-1’), 4.61 (1H, d, *J*_AB_ 6.8 Hz, OCH_2_O), 4.66 (1H, d, *J*_AB_ 6.8 Hz, OCH_2_O), 4.67 (1H, d, *J*_AB_ 6.8 Hz, OCH_2_O), 4.86 (1H, d, *J*_AB_ 6.8 Hz, OCH_2_O), 5.07 (1H, s, H-3), 6.10 (1H, s (br), NH), 7.35-7.45 (6H, m, Ar), 7.60-7.70 (4H, m, Ar); δ_C_ (CDCl_3_, 100.6 MHz) 19.2 (Si-C), 26.8 ((CH_3_)_3_), 55.8 (OCH_3_), 56.2 (OCH_3_), 58.7 (OCH_3_), 58.8 (C-5), 63.3 (C-4’), 71.7 (C-1’), 78.4 (C-3’), 78.9 (C-2’), 94.8 (C-3), 96.7 (OCH_2_O), 98.9 (OCH_2_O), 127.7, 127.7, 129.8, 129.8, 133.1, 133.1, 135.6, 135.6 (Ar), 173.5 (C-4), 177.5 (C-2).

1’*R*, 2’*S*, 3’*S*, 5*S*, 3-Bromo-5-[4’-*tert*-butyldiphenylsilyloxy-1’-hydroxy-2’,3’-bis-methoxymethoxy]-butyl-4,4-dimethoxy-pyrrolidin-2-one **11**

To a solution of lactam **10** (0.60 g, 1.1 mmol) in methanol at -20°C, was added bromine (1 M in dichloromethane, 1.5 ml, 1.5 mmol, 1.5 eq). The reaction mixture was stirred at -20°C for 30 minutes and then quenched with cold saturated sodium carbonate (50 ml) at -20°C and concentrated under reduced pressure. The product was extracted with ethyl acetate (3 x 100 ml). The combined organic layers were dried over magnesium sulfate and concentrated under reduced pressure to give an oil (0.90 g). The crude mixture was purified on silica-gel (65 g) using ethyl acetate as eluent to give bromide **11** (0.70 g, 1.0 mmol, 91%), as a 4 : 1 mixture of diastereomers.

Major: **11** [α]_D_ –5.75 (c 2.0, CH_2_Cl_2_); ([M+H]^+^ Found 670.2026, C_30_H_45_NO_9_SiBr requires 670.2047.); υ_max_ /cm^-1^ 3526 (N-H), 3406 (O-H), 3071, 3051 (C-H arom), 2950, 2933, 2892, 2857, 2824 (C-H aliph), 1726 (C=O), 1114 (C-O), 614 (C-Br); δ_H_ (CDCl_3_, 400 MHz) 1.06 (9H, s, *t*-Bu), 3.23 (3H, s, OCH_3_), 3.37 (3H, s, OCH_3_), 3.42 (3H, s, OCH_3_), 3.45 (3H, s, OCH_3_), 3.78-4.01 (5H, m, H-2’, H-3’, H-4’ and H-5), 4.02 (1H, s, H-3), 4.52 (1H, m, H-1’), 4.54 (1H, d, *J*_AB_ 6.6 Hz, OCH_2_O), 4.58 (1H, d, *J*_AB_ 6.6 Hz, OCH_2_O), 4.68 (1H, d, *J*_AB_ 6.6 Hz, OCH_2_O), 4.93 (1H, d, *J*_AB_ 6.6 Hz, OCH_2_O), 7.02 (1H, s (br), NH), 7.30-7.50 (6H, m, Ar), 7.60-7.70 (4H, m, Ar); δ_C_ (CDCl_3_, 100.6 MHz) 19.2 (Si-C), 26.9 ((CH_3_)_3_), 45.1 (C-3), 50.5 (OCH_3_), 51.3 (OCH_3_), 55.7 (OCH_3_), 55.9 (OCH_3_), 57.5 (C-5), 63.3 (C-4’), 69.2 (C-1’), 78.3 (C-3’), 78.4 (C-2’), 96.4 (OCH_2_O), 99.0 (OCH_2_O), 105.4 (C-4), 127.7, 127.8, 129.7, 129.7, 133.4, 133.5, 135.6, 135.7 (Ar), 170.2 (C-2).

1’*R*, 2’*S*, 3’*S*, 5*S*, 5-(4’-*tert*-Butyldiphenylsilyloxy-1’-hydroxy-2’,3’-bis-methoxymethoxy)-butyl-4,4-dimethoxy-pyrrolidin-2-one **12**

To a solution of bromide **11** (0.70 g, 1.0 mmol) in tetrahydrofuran / aqueous ammonium chloride / methanol (2 : 2 : 1, 50 ml), was added zinc powder (0.34 g, 5.0 mmol, 5 eq). The reaction mixture was stirred at room temperature for 30 minutes, before being filtered through Celite® and the precipitate thoroughly rinsed with ethyl acetate. Saturated sodium bicarbonate (50 ml) was added to the filtrate and the whole concentrated under reduced pressure. The product was extracted with ethyl acetate (3 x 100 ml). The combined organic layers were dried over magnesium sulfate and concentrated under reduced pressure to yield an oil (0.67 g), which was purified on silica-gel (55 g) using ethyl acetate as the eluent to give ketal **12** (0.52 g, 0.92 mmol, 90%).

M.p. 92-94°C (from ethyl acetate-hexane); [α]_D_ –24.7 (c 2.0, CH_2_Cl_2_); (Found: C, 60.97; H, 7.41; N, 2.56%. C_30_H_45_NO_9_Si requires C, 60.89; H, 7.66; N, 2.37%); [M+H]^+^ Found 592.2912, C_50_H_46_NO_9_ requires 592.2942; υ_max_ /cm^-1^ 3532 (N-H), 3352 (O-H), 3070, 3053, (C-H arom), 2935, 2893, 2858 (C-H aliph), 1713 (C=O), 1113, 1067 (C-O ether); δ_H_ (CDCl_3_, 300 MHz) 1.06 (9H, s, *t*-Bu), 2.44 (1H, d, *J* 16.4 Hz, H-3), 2.55 (1H, d, *J* 16.4 Hz, H-3), 3.27 (3H, s, OCH_3_), 3.30 (3H, s, OCH_3_), 3.33 (3H, s, OCH_3_), 3.40 (1H, s, OH), 3.43 (3H, s, OCH_3_), 3.84-4.03 (6H, m, H-1’, H-2’, H-3’, H-4’ and H-5), 4.65 (2H, s, OCH_2_O), 4.66 (1H, d, *J*_AB_ 6.2 Hz, OCH_2_O), 4.96 (1H, d, *J*_AB_ 6.2 Hz, OCH_2_O), 6.75 (1H, s(br), NH), 7.30-7.50 (6H, m, Ar), 7.60-7.70 (4H, m, Ar); δ_C_ (CDCl_3_, 75.5 MHz) 19.2 (Si-C), 26.7 ((CH_3_)_3_), 40.3 (C-3), 48.8 (OCH_3_), 51.2 (OCH_3_), 55.7 (OCH_3_), 56.0 (OCH_3_), 58.0 (C-5), 63.61 (C-4’), 70.1 (C-1’), 78.7 (C-3’), 79.0 (C-2’), 96.7 (OCH_2_O), 99.0 (OCH_2_O), 107.1 (C-4), 127.7, 127.7, 129.7, 129.7, 133.8, 133.3, 135.5, 135.6 (Ar), 172.1 (C-2).

1’*R,* 2’*S*, 3’*S*, 5*S*, 2-[1’-*tert*-Butoxycarbonyloxy-4’-*tert*-butyldiphenyl-silyloxy-2’,3’-bis-methoxymethoxy]-butyl-3,3-dimethoxy-5-oxo-pyrrolidine-1-carboxylic acid *tert*-butyl ester **13**

To a solution of lactam **12** (1.50 g, 2.5 mmol) in dry tetrahydrofuran (305 ml), were added di-*tert*-butyl dicarbonate (2.20 g, 10 mmol, 4 eq) and dimethylaminopyridine (0.17 g, 1.4 mmol, 0.5 eq). The reaction mixture was stirred at room temperature for 16h, after which it was concentrated under reduced pressure to yield an oil. The crude product was purified on silica-gel (100 g) using ethyl acetate / hexane (3 : 7) as eluent to give **13** (1.60 g, 2.0 mmol, 80%).

M.p. 158-159°C (from ethyl acetate-petroleum ether); [α]_D_ + 2.3 (*c* 3, CHCl_3_), (Found: C, 60.44; H, 7.70; N, 1.36%. C_40_H_61_NO_13_Si requires C, 60.66; H, 7.76; N, 1.77%); ([M+Na]^+^ Found 814.3819, C_40_H_61_NO_13_SiNa requires 814.3810.); υ_max_ /cm^-1^ 3072 (C-H arom), 2979 2958, 2935, 2892, 2857 (C-H aliph), 1752 (O-C=O), 1716 (N-Boc-C=O), 1635 (C=O amide), 1133, 1106 (C-O ether); δ_H_ (CDCl_3_, 400 MHz) 1.07 (9H, s, *t*-Bu), 1.42 (9H, s, *t*-Bu), 1.50 (9H, s, *t*-Bu), 2.49 (1H, d, *J* 16.2 Hz, H-4), 3.02 (1H, d, *J* 16.2 Hz, H-4), 3.24 (3H, s, OCH_3_), 3.26 (3H, s, OCH_3_), 3.29 (3H, s, OCH_3_), 3.32 (3H, s, OCH_3_), 3.94 (1H, dd, *J* 9.8, 6.2 Hz, H-4’), 4.00 (1H, td, *J* 6.2, 1.9 Hz, H-3’), 4.10 (1H, dd, *J* 9.8, 6.2 Hz, H-4’), 4.45 (1H, dd, *J* 9.0, 1.9 Hz, H-2’), 4.64 (1H, d, *J* 2.0 Hz, H-2), 4.70 (1H, d, *J* 6.6 Hz, OCH_2_O), 4.70 (2H, s, OCH_2_O), 4.84 (1H, d, *J* 6.6 Hz, OCH_2_O), 5.41 (1H, d, *J* 9.0, 2.0 Hz, H-1’), 7.30-7.50 (6H, m, Ar) 7.60-7.70 (4H, m, Ar); δ_C_ (CDCl_3_, 100.6 MHz) 19.2 (Si-C), 26.9 (SiC(*C*H_3_)_3_), 27.7 (OCO_2_C(*C*H_3_)_3_), 28.0 (OCO_2_C(*C*H_3_)_3_), 43.6 (C-4), 48.7 (OCH_3_), 51.0 (OCH_3_), 55.7 (OCH_3_), 56.1 (OCH_3_), 59.9 (C-2), 63.1 (C-4’), 76.0 (C-1’), 77.2 (C-2’), 77.5 (C-3’), 82.6 (O*C*(CH_3_)_3_), 83.3 (O*C*(CH_3_)_3_), 96.7 (OCH_2_O), 98.3 (OCH_2_O), 102.6 (C-3), 127.6, 127.7, 129.7, 129.7, 133.4, 133.5, 135.5, 135.5 (Ar), 149.7 (CO Boc), 153.2 (CO Boc), 169.2 (C-5);

1’*R*, 2’*S*, 3’*S*, 5*S*, 2-[1’-*tert*-Butoxycarbonyloxy-4’-(*tert*-butyl-diphenylsilyloxy)-2’,3’-bis-methoxymethoxy]-butyl-3,3-dimethoxy-5-hydroxy-pyrrolidine-1-carboxylic acid *tert*-butyl ester **14**

To a solution of carbamate **13** (0.70 g, 0.88 mmol) in dry tetrahydrofuran (50 ml) at -78°C was added DIBAL (1.5 M in toluene, 2.35 ml, 3.5 mmol, 4 eq) drop-wise. The reaction mixture was stirred over two hours between -60°C and -50°C. The reaction mixture was quenched with a saturated solution of sodium acetate at -78°C and a mixture of ammonium chloride and ethyl acetate (1 : 3) was added. The mixture was allowed to warm to room temperature after which the mixture was filtered through Celite® and the filtrate extracted three times with ethyl acetate and washed with ammonium chloride. The combined organic layer was dried over magnesium sulfate and concentrated under reduced pressure to yield an oil (0.80 g). The crude product was purified on silica-gel (50 g) using ethyl acetate / petroleum ether (3 : 7) as an eluent to give lactol **14** (0.60 g, 0.76 mmol, 86%).

M.p. 118-119°C (from Ethyl Acetate / Petroleum Ether); [α]_D_ -3.1 (*c* 3, CHCl_3_), [M+Na]^+^ Found 816.3928 C_40_H_63_NO_13_SiNa requires 816.3966; υ_max_ /cm^-1^ 3445 (O-H), 2978 (C-H arom), 2858 (C-H aliph), 1749 (C=O), 1634 (C=O), 1108 (C-O). The ^1^H and ^13^C NMR spectra were too complex to analyze, because of stereogenic elements (*N*-Boc and lactol).

1’*R*, 2’*S*, 3’*S*, 5*S*, 2-[1’-*tert*-Butoxycarbonyloxy-4’-(tert-butyl-diphenyl-silyloxy)-2’,3’-bis-methoxymethoxy]-butyl-3,3-dimethoxy-pyrrolidine-1-carboxylic acid *tert*-butyl ester **15**

To a solution of lactol **14** (400 mg, 0.51 mmol) in dry dichloromethane (20 ml) at -78°C, were added triethylsilane (0.16 ml, 1.02 mmol, 2 eq) and borontrifluoride etherate (0.13 ml, 1.02 mmol, 2 eq). The reaction mixture was stirred for 1hr at -78°C. Triethylamine (0.21 ml, 1.53 mmol, 3 eq) was added followed by saturated sodium carbonate and the reaction mixture was allowed to warm up to room temperature with stirring. The crude product was extracted three times with ethyl acetate and the combined organic layer was dried over magnesium sulfate and concentrated to yield a residue (750 mg). The product was purified on silica-gel (40 g) using ethyl acetate / petroleum ether (1 : 4) as eluent to give **15** (360 mg, 0.46 mmol, 91%) as an oil and as a mixture of *N*-Boc rotamers.

[M+H]^+^ Found 778.4216, C_40_H_64_NO_9_Si requires 778.4198; υ_max_ /cm^-1^ 3072, 3051 (C-H arom), 2963, 2933, 2895, 2857 (C-H aliph), 1750 (O-C=O), 1697 (N-C=O), 1162, 1109 (C-O);

Major-δ_H_ (CDCl_3_, 400 MHz) 1.06 (9H, s, *t*-Bu), 1.40 (9H, s, *t*-Bu), 1.43 (9H, s, *t*-Bu), 2.02 (1H, m, H-4), 2.32 (1H, m, H-4), 3.19 (3H, s, OCH_3_), 3.22 (3H, s, OCH_3_), 3.28 (3H, s, OCH_3_) 3.32 (2H, m, H-5), 3.35 (3H, s, OCH_3_), 3.99 (3H, m, H-3’, H-4’), 4.27 (1H, d, *J* 3.2 Hz, H-2), 4.53 (1H, t, *J* 8.0 Hz, H-2’), 4.72 (4H, m, OCH_2_O), 5.30 (1H, dd, *J* 8.0, 3.2 Hz, H-1’), 7.30-7.50 (6H, m, Ar) 7.60-7.70 (4H, m, Ar); δ_C_ (CDCl_3_, 100.6 MHz) 19.2 (Si-C), 26.8 ((CH_3_)_3_), 27.7 (OCO_2_C(*C*H_3_)_3_), 28.4 (OCO_2_C(*C*H_3_)_3_), 32.8 (C-4), 43.6 (C-5), 49.1 (OCH_3_), 50.5 (OCH_3_), 55.8 (OCH_3_), 56.0 (OCH_3_), 57.4 (C-2), 63.7 (C-4’), 76.6 (C-1’), 77.5 (C-3’), 78.2 (C-2’), 79.2 (O*C*(CH_3_)_3_), 81.3 (O*C*(CH_3_)_3_), 97.4 (OCH_2_O), 98.4 (OCH_2_O), 108.4 (C-3), 127.6, 127.7, 129.7, 129.7, 133.4, 133.5, 135.5, 135.5 (Ar), 149.7 (CO), 153.2 (CO).

Minor-δ_H_ (CDCl_3_, 400 MHz) 1.06 (9H, s, *t*-Bu), 1.44 (9H, s, *t*-Bu), 1.50 (9H, s, *t*-Bu), 2.02 (1H, m, H-4), 2.32 (1H, m, H-4), 3.19 (3H, s, OCH_3_), 3.22 (3H, s, OCH_3_), 3.28 (3H, s, OCH_3_), 3.32 (2H, m, H-5), 3.35 (3H, s, OCH_3_), 3.99 (3H, m, H-3’, H-4’), 4.19 (1H, d, *J*  3.2 Hz, H-2), 4.33 (1H, t, *J* 8.0 Hz, H-2’), 4.72 (3H, m, OCH_2_O), 4.89 (1H, d, *J* 6.4 Hz, OCH_2_O), 5.38 (1H, dd, *J* 8.0, 3.2 Hz, H-1’), 7.30-7.50 (6H, m, Ar) 7.60-7.70 (4H, m, Ar); δ_C_ (CDCl_3_, 100.6 MHz) 19.2 (Si-C), 26.8 ((CH_3_)_3_), 27.8 (OCO_2_*C*(CH_3_)_3_), 28.4 (OCO_2_C(*C*H_3_)_3_), 32.0 (C-4) 43.6 (C-5), 48.5 (OCH_3_), 50.5 (OCH_3_), 55.4 (OCH_3_), 56.1 (OCH_3_), 58.0 (C-2), 63.5 (C-4’), 76.8 (C-1’), 77.6 (C-3’), 78.2 (C-2’), 80.3 (O*C*(CH_3_)_3_), 81.7 (O*C*(CH_3_)_3_), 96.4 (OCH_2_O), 98.6 (OCH_2_O), 109.0 (C-3), 127.6, 127.7, 129.7, 129.7, 133.4, 133.5, 135.5, 135.5 (Ar), 149.7 (CO), 153.2 (CO).

1’*R*, 2’*S*, 3’*S*, 5*S*, 2-[1’-*tert*-Butyloxycarbonyloxy-4’-hydroxy-2’,3’-bis-methoxymethoxy]-butyl-3,3-dimethoxy-pyrrolidine-1-carboxylic acid *tert*-butyl ester **16**

To a solution of amine **15** (600 mg, 0.77 mmol) in tetrahydrofuran (20 ml) at -20°C was added tetrabutylammonium fluoride (1.16 ml, 1.16 mmol, 1.5 eq). The reaction mixture was allowed to warm up to 10°C and stirred for five days at 10°C. The reaction mixture was quenched with ammonium chloride and extracted three times with ethyl acetate. The combined organic layers were dried over magnesium sulfate and concentrated to yield a residue. The residue was purified on silica-gel (50 g), using ethyl acetate / petroleum ether mixtures (2 : 3 to 1 : 1) to yield **16** (335 mg, 0.62 mmol, 81%).

M.p. 128-130°C (from Ethyl Acetate / Hexane); [α]_D_ -7.5 (*c* 1, CH_2_Cl_2_); (Found: C, 53.52; H 8.39; N, 2.46%. C_24_H_45_NO_12_ requires C, 53.42; H, 8.41; N, 2.60%.); [M+H] 540.2997, C_24_H_46_NO_12_ requires 540.3020; υ_max_ /cm^-1^ 3475 (O-H), 2976, 2944, 2898, 2837 (C-H aliph), 1750 (O-C=O), 1697 (N-C=O), 1163, 1108 (C-O);

Major- δ_H_ (CDCl_3_, 400 MHz) 1.42 (9H, s, *t-*Bu), 1.45 (9H, s, *t-*Bu),1.74 (1H, s, OH), 2.01 (1H, m, H-4), 2.62 (1H, m, H-4), 3.22 (3H, s, OCH_3_), 3.27 (3H, s, OCH_3_), 3.31 (2H, m, H-5), 3.39 (3H, s, OCH_3_), 3.44 (3H, s, OCH_3_), 3.81 (3H, m, H-3’ H-4’), 4.21 (1H, d, *J* 2.4 Hz, H-2), 4.28 (1H, t, *J* 8.0 Hz, H-2’), 4.59 (1H, d, *J* 6.9 Hz, OCH_2_O), 4.80 (3H, m, OCH_2_O), 5.40 (1H, m, H-1’); δ_C_ (CDCl_3_, 100.6 MHz) 27.8 (OCO_2_C(*C*H_3_)_3_), 28.4 (OCO_2_C(*C*H_3_)_3_), 33.0 (C-4), 43.7 (C-5), 48.9 (OCH_3_), 50.5 (OCH_3_), 55.9 (OCH_3_), 56.2 (OCH_3_), 57.3 (C-2), 62.5 (C-4’), 76.0 (C-1’), 77.0 (C-2’), 79.5 (C-3’), 81.0 (O*C*(CH_3_)_3_), 81.5 (O*C*(CH_3_)_3_), 97.6 (OCH_2_O), 98.4 (OCH_2_O), 108.4 (C-3),153.1 (CO), 154.2 (CO);

Minor- δ_H_ (CDCl_3_, 400 MHz) 1.44 (9H, s, *t-*Bu), 1.50 (9H, s, *t-*Bu), 2.01 (1H, m, H-4), 2.62 (1H, m, H-4), 3.20 (3H, s, OCH_3_), 3.27 (3H, s, OCH_3_), 3.31 (2H, m, H-5), 3.41 (3H, s, OCH_3_), 3.42 (3H, s, OCH_3_), 3.81 (3H, m, H-3’, H-4’ ), 4.08 (1H, d, *J* 1.8 Hz, H-2), 4.28 (1H, t, *J* 8.0 Hz, H-2’), 4.70 (1H, d, *J* 6.9 Hz, OCH_2_O), 4.80 (3H, m, OCH_2_O), 5.40 (1H, m, H-1’); δ_C_ (CDCl_3_, 100.6 MHz) 27.8 (OCO_2_C(*C*H_3_)_3_), 28.5 (OCO_2_C(*C*H_3_)_3_), 32.0 (C-4), 43.7 (C-5), 48.6 (OCH_3_), 50.5 (OCH_3_), 55.9 (OCH_3_), 56.4 (OCH_3_), 57.7 (C-2), 62.6 (C-4’), 76.0 (C-1’), 78.0 (C-2’), 80.4 (C-3’), 80.7 (O*C*(CH_3_)_3_), 81.9 (O*C*(CH_3_)_3_), 97.0 (OCH_2_O), 98.6 (OCH_2_O), 108.9 (C-3), 153.0 (CO), 154.2 (CO).

1’*R*, 2’*S*, 3’*S*, 5*S*, 2-[1’-(*tert*-Butoxycarbonyloxy)-2’,3’-bis*-*methoxymethoxy-4’-(*p-*toluene-sulfonyloxy)]-butyl-3,3-dimethoxy-pyrrolidine-1-carboxylic acid *tert*-butyl ester **17**

To a solution of alcohol **16** (60 mg, 0.12 mmol) in dry dichloromethane (5 ml) at 0°C, were added tosyl chloride (30 mg, 0.18 mmol, 1.5 eq), triethylamine (0.04 ml, 0.24 mmol, 2 eq) and DMAP (a catalytic amount). The reaction mixture was stirred at room temperature. Every 4 hrs, an extra 1.5 equivalents of tosyl chloride and 2 equivalents of triethylamine were added until the reaction had gone to completion (48 hrs). The reaction mixture was poured over an ice/water mixture and the crude product was extracted with ethyl acetate (3 x 20 ml). The combined organic layers were dried over magnesium sulfate and concentrated to yield a residue, which was purified on silica-gel (5 g) using ethyl acetate / petroleum ether (2 : 3) as eluent to yield **17** (75 mg, 100%, 0.12 mmol) as a 2:1 mixture of *N*-Boc rotamers by ^1^H NMR.

M.p. 135-136°C (from ethyl acetate / petroleum ether); [α]_D_ +0.8 (*c* 1.5 CH_2_Cl_2_); (Found: C, 53.61; H, 7.43; N, 1.90; S, 4.47%. C_31_H_51_NO_14_S requires C, 53.67; H, 7.41; N, 2.02; S, 4.62%.); ([M+H]^+^ Found 694.3110, C_31_H_52_NO_14_S requires 694.3109.); υ_max_ /cm^-1^ 2976, 2898, 2828 (C-H aliph), 1749 (O-C=O), 1690 (N-C=O), 1367 (S=O), 1162, 1108 (C-O).

Major- δ_H_ (CDCl_3_, 400 MHz) 1.41 (9H, s, *t*-Bu), 1.43 (9H, s, *t*-Bu), 1.96 (1H, m, H-4), 2.24 (1H, m, H-4), 2.40 (3H, s, CH_3_), 3.17 (3H, s, OCH_3_), 3.22 (3H, s, OCH_3_), 3.25 (2H, m, H-5), 3.25 (3H, s, OCH_3_), 3.35 (3H, s, OCH_3_), 4.11 (2H, m, H-2’, H-3’), 4.21 (1H, d, *J* 4.2 Hz, H-2), 4.25-.4.40 (2H, m, H-4’), 4.56 (1H, m, OCH_2_O), 4.62-4.74 (3H, m, OCH_2_O) 5.14 (1H, dd, *J* 4.2, 7.6 Hz, H-1’), 7.29 (2H, d, *J* 7.8 Hz, Ar), 7.81 (2H, d, *J* 7.8 Hz, Ar); δ_C_ (CDCl_3_, 100.6 MHz) 21.6 (CH_3_), 27.8 (OCO_2_C(*C*H_3_)_3_), 28.4 (OCO_2_C(*C*H_3_)_3_), 32.6 (C-4), 43.6 (C-5), 48.9 (OCH_3_), 50.5 (OCH_3_), 56.0 (OCH_3_), 56.0 (OCH_3_), 57.2 (C-2), 70.0 (C-4’), 74.7 (C-1’), 75.4 (C-2’), 76.0 (C-3’), 79.5 (O*C*(CH_3_)_3_), 81.7 (O*C*(CH_3_)_3_), 96.8 (OCH_2_O), 98.4 (OCH_2_O), 108.2 (C-3),127.9, 128.1, 129.7, 133.2 (Ar), 153.1 (CO), 154.2 (CO);

Minor- δ_H_ (CDCl_3_, 400 MHz) 1.41 (9H, s, *t*-Bu), 1.53 (9H, s, *t-*Bu), 1.96 (1H, m, H-4), 2.21 (1H, m, H-4), 2.40 (3H, s, CH_3_), 3.17 (3H, s, OCH_3_), 3.22 (3H, s, OCH_3_), 3.25 (2H, m, H-5), 3.25 (3H, s, OCH_3_), 3.33 (3H, s, OCH_3_), 4.11 (2H, m, H-2’, H-3’), 4.25-.4.40 (3H, m, H-4’, H-2), 4.54 (1H, m, OCH_2_O), 4.62-4.74 (3H, m, OCH_2_O) 5.22 (1H, m, H-1’), 7.31 (2H, d, *J* 7.8 Hz, Ar), 7.77 (2H, d, *J* 7.8 Hz, Ar); δ_C_ (CDCl_3_, 100.6 MHz) 21.6 (CH_3_), 27.8 (OCO_2_C(*C*H_3_)_3_), 28.4 (OCO_2_C(*C*H_3_)_3_), 32.0 (C-4), 43.6 (C-5), 48.9 (OCH_3_), 50.5 (OCH_3_), 56.0 (OCH_3_), 56.0 (OCH_3_), 57.9 (C-2), 69.2 (C-4’), 74.7 (C-1’), 75.4 (C-2’), 76.0 (C-3’), 79.5 (O*C*(CH_3_)_3_), 81.7 (O*C*(CH_3_)_3_), 96.6 (OCH_2_O), 98.5 (OCH_2_O), 108.8 (C-3), 127.9, 128.1, 129.7, 133.2 (Ar), 153.1 (CO), 154.2 (CO);

6*S*, 7*S*, 8*R*, 8a*S* 1,1-Dimethoxy-octahydroindolizidine-6,7,8-triol **18**

A solution of tosylate **17** (40 mg, 0.058 mmol) in dry dichloromethane and trifluoroacetic acid (0.75 ml: 0.25 ml), (3 :1) was stirred at 0°C for 2 hrs. The excess dichloromethane and trifluoroacetic acid were removed under vacuum for 45 minutes. The residue was dissolved in dry dichloromethane (2 ml) and cooled to 0°C and diisopropylethylamine (1 ml) was added. The reaction mixture was stirred at room temperature for 18h. The reaction mixture was concentrated *in vacuo* and redissolved in methanol. Potassium carbonate was added and the mixture was stirred for 30 minutes. The mixture was filtered through Celite and the Celite rinsed thoroughly with methanol. The filtrate was concentrated to yield a residue (50 mg), which was purified on silica-gel (10 g) using ethyl acetate, followed by methanol / ethyl acetate (9 : 1 to 4 : 1) with three drops of ammonia to yield **18** as a tosylate salt (13 mg, 0.039 mmol, 56%).

δ_H_ (CD_3_OD, 400 MHz) 1.90 (1H, m, H-2), 2.10 (1H, m, H-2), 2.35 (3H, s, CH_3_), 3.05 (1H, m, H-3), 3.15 (1H, m, H-3) 3.35 (3H, s, OCH_3_), 3.36 (3H, s, OCH_3_), 3.57 (1H, d, *J* 6.4 Hz, H-8a), 3.88 (1H, d, *J* 9.6 Hz, H-5), 4.04 (1H, dd, *J* 9.6 and 3.4 Hz, H-5), 4.32 (1H, d, *J* 3.4 Hz, H-6), 4.55 (1H, d, *J* 3.6 Hz, H-7), 4.68 (1H, dd, *J* 6.4 and 3.6 Hz, H-8), 7.22 (2H, d, Ar), 7.70 (2H, d, Ar); δ_C_ (CD_3_OD, 100.6 MHz) 21.3 (Ts(CH_3_)), 34.6 (C-2), C-3, OCH_3_ both under CD_3_OD, 51.6 (OCH_3_), 70.0 (C-8a), 75.8 (C-6), 77.0 (C-5), 83.5 (C-8), 91.8 (C-7),122.1 (C-1), 127.0, 129.8, 141.7, 143.6 (Ar).
